# Returning to work in lung cancer survivors—a multi-center cross-sectional study in Germany

**DOI:** 10.1007/s00520-020-05886-z

**Published:** 2020-11-19

**Authors:** Humayra Rashid, Martin Eichler, Marlene Hechtner, Emilio Gianicolo, Beatrice Wehler, Roland Buhl, Heinz Schmidberger, Jan A. Stratmann, Bernhard Gohrbandt, Cornelius Kortsik, Ursula Nestle, Hubert Wirtz, Maria Blettner, Susanne Singer

**Affiliations:** 1 Division of Epidemiology and Health Services Research, Institute of Medical Biostatistics, Epidemiology and Informatics, University Medical Center, Johannes Gutenberg University Mainz, Obere Zahlbacher Straße 69, 55131 Mainz, Germany; 2 University Cancer Center Mainz, Mainz, Germany; 3 National Center for Tumor Diseases (NCT/ UCC), University Hospital Carl Gustav Carus, Technische Universität Dresden, Dresden, Germany; 4 Institute of Clinical Physiology of the Italian National Research Council, Lecce, Italy; 5 Department of Clinical Oncology, Saarland University Medical Center, Homburg, Germany; 6 Pulmonary Department, University Medical Center, Johannes Gutenberg University Mainz, Mainz, Germany; 7 Department of Radiation-Oncology, University Medical Center, Johannes Gutenberg University Mainz, Mainz, Germany; 8 Department of Internal Medicine, University Hospital Frankfurt, Goethe University, Frankfurt, Germany; 9 Department of Thoracic Surgery, Katholisches Klinikum Mainz, Mainz, Germany; 10 Department of Pneumology, Katholisches Klinikum Mainz, Mainz, Germany; 11 Department of Radiation Oncology, Maria Hilf Hospital Moenchengladbach, Moenchengladbach, Germany; 12 Department of Radiation Oncology, University Medical Center Freiburg, Freiburg, Germany; 13 Department of Pneumology, University Medical Center Leipzig, Leipzig, Germany

**Keywords:** Returning to work, Employment, Lung cancer, Early retirement

## Abstract

**Purpose:**

To investigate the work situation of lung cancer survivors and to identify the factors associated with their returning to work.

**Methods:**

Descriptive analysis and logistic regression were used to evaluate study population characteristics and independent factors of subsequently returning to work. To analyze time to return to work, Cox regression was used.

**Results:**

The study sample included 232 lung cancer survivors of working age from 717 enrolled participants in the multi-center cross-sectional LARIS (Quality of Life and Psychosocial Rehabilitation in Lung Cancer Survivors) study. About 67% of the survivors were not employed during the survey. More than 51% of the survivors who were employed before their illness did not return to their work. The survivors who had returned to their careers were younger, associated with higher household income, lower fatigue score, and stable relationship and vocational training. Patients who received social service counseling showed a higher chance of regaining their career.

**Conclusions:**

Lung cancer survivors were found to be associated with a high risk of unemployment and very low professional reintegration after interruption due to illness. More comprehensive studies are needed to support lung cancer survivors and targeting of patients in need of special attention in rehabilitation that would benefit from the findings in the present study.

**Supplementary Information:**

The online version contains supplementary material available at 10.1007/s00520-020-05886-z.

## Background

As treatment for lung cancer advances, the number of survivors is increasing [1]. Immunotherapy with immune checkpoint inhibitors (ICIs) against programmed cell death 1 (PD-1/L1) axis has to be proven to aid the probabilities of long-term survival for advanced lung cancer patients [2]. Consequently, concerns regarding improving survivors’ quality of life and functioning are drawing more attention. Among various aspects of successful rehabilitation, returning to work is important as it is related to self-efficacy, belonging to the society and financial security [3]. Unfortunately, among lung cancer patients, there is a high risk of unemployment and early retirement compared to the general population [4]. In a Norwegian and a Finnish cohort, the relative risk of unemployment was respectively 63% and 45% higher among lung cancer participants than in cancer-free patients [4]. Higher risk of unemployment was found in both gender [4].

Returning to work (RTW) is defined as the recovery of one’s performance ability at the workplace after surviving an illness [4]. It can be viewed as regaining a normal life [5, 6]. Despite its importance, studies focusing on lung cancer survivor’s occupational reintegration, both national and international, are limited and have included relatively small numbers of participants to date. Previous studies have reported that lung cancer survivors were less likely to be employed than the non-cancer group, and they faced early job loss [7, 8]. Even in comparison to other cancer patients, a lower employment status was seen among lung cancer patients [9, 10]. Moreover, lung cancer seemed to impact persistently on employment years after the diagnosis [11]. In a 2013 study including different types of cancer, Mehnert et al. reported that 43% of the lung cancer survivors in their sample did not return to their workplace, which was the second highest percentage of unemployment in the entire study [12]. Immediately after the end of inpatient rehabilitation, only 10% of lung cancer survivors had returned to their workplace [12]. However, these results were based on only 23 lung cancer survivors [12].

A German cohort study stated 15% of lung cancer patients under the age of 55 years were retired 15 months after diagnosis [13]. However, only a few lung cancer patients were included: of 491 cancer patients, 20 had been diagnosed with lung cancer.

Many studies have highlighted several factors in relation to returning to work among cancer survivors. High job requirements, no cancer progression, intention to returning to work, no baseline sick leave absence, unproblematic social interactions, occupation, stable relationship, absence of comorbidities, cancer remission, time since last treatment, younger age (< 50 years), a higher education level, high income, no chemotherapy or combination therapy, male sex, low fatigue, higher value of work, job self-efficacy, and perceived work ability emerged as significant factors facilitating returning to work [5, 9, 12, 14–16]. Furthermore, heavy work or manual work, depression, vocational training, negative effects of treatment modalities, invasive surgeries, and more extensive diseases were associated with more limitations in working abilities [10, 17]. Early identification of these risk factors for not returning to work may help health professionals to identify survivors at a higher risk of not returning to work and so allow for early supportive intervention, to help lung cancer survivors to resume their work capacities and maintain financial security.

Lung cancer causes physical and psychological limitations and thus restricts professional performance in workplace [18]. The side effects of cancer therapies also have an impact on the individual’s work ability to handle the workload [18]. In addition, frequent absences due to therapy or rehabilitation interrupt work processes [18]. According to data from the German Federal Pension Insurance, in the year 2012, a major proportion of lung cancer participants (65% of working men and 57% of women) were only able to work up to 3 h per day after receiving medical rehabilitation [19]. This situation accentuates a major need to study the workplace circumstances and the factors responsible for reduced working capabilities among lung cancer survivors to aid their professional life and, hence, achieve a better quality of life.

Social service counseling (SSC) by the social worker in cancer care is the integral and mandatory part of the German Cancer Care Centers [20]. Providing SSC to the cancer patients is a cardinal requirement of the German Cancer Society for certification. Social workers involving in providing SSC play very important roles in contributing occupational rehabilitation and in supporting the employees with legal assistance when they feel discriminated at their work place due to their disease [20]. So, whether this counseling service has the role in the regaining career of the lung cancer survivors at the German context draws attention to analyze thoroughly.

Therefore, the current study aims to assess the work situation and factors of returning to work of lung cancer survivors. The objectives of the study are:

- To investigate the number of lung cancer survivors who were diagnosed at working age, are now (> 1 year after diagnosis) employed, (early) retired, or unemployed and the number of the survivors who worked before the illness has returned to work after the illness and to find out how many months after diagnosis did the resumption of employment or retirement begin
- To see if the patients who receive social service counseling (SSC) return to work faster than those who do not receive
- To examine the number of unemployed survivors of working age > 1 year after diagnosis who would like to return to work
- To study the factors influenced returning to work

## Participants and methods

### Study design and study population

**Fig. 1:**
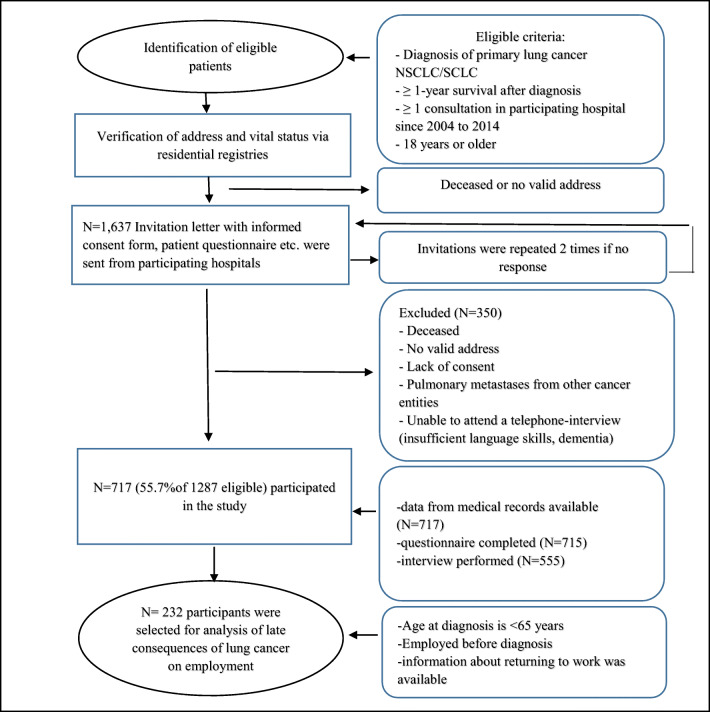
Flow chart of data collection within LARIS study. NSCLC, non-small cell lung carcinoma; SCLC, small cell lung carcinoma; CATI, computer-assisted telephone interview

A total of 717 lung cancer survivors were enrolled in the multi-center cross-sectional LARIS (Quality of Life and Psychosocial Rehabilitation in Lung Cancer Survivors) study with retrospectively self-reported data on returning to work [21, 22]. The study design and data collection have been described in previous papers [21, 22]. In brief, the participants were recruited from six institutions in Germany (university hospitals in Mainz, Frankfurt, Leipzig, Freiburg, and Homburg, and the Catholic Hospital Mainz) between February 2015 and September 2016. The inclusion criteria were the participants who (1) had a diagnosis of primary lung cancer non-small cell lung carcinoma (NSCLC) or small cell lung carcinoma (SCLC), (2) have survived 1 year or longer beyond diagnosis, (3) had at least one admission (e.g., diagnosis, treatment, or after-care visit) to a participating study center during 2004 to 2014, and (4) were 18 years of age or older at diagnosis [21, 22]. To analyze the occupational status, the participants who were less than 65 years old at the time of diagnosis, whose information about returning to work was available and who had worked before the diagnosis, were included in this current analysis (Fig. 1).

Personal invitation letters (*N* = 1637) along with study questionnaires and a consent form were sent to the patient’s verified addresses by the participating hospitals [21, 22]. Then, after receiving written informed consent from the participants, a corresponding computer-assisted telephone interview (CATI) or a face to face interview was conducted with each participant [21]. The participants who did not reply were contacted again within 2 months with reminder letters, invitation documents, and the questionnaires.

Approvals were obtained from the Ethics Committee of the Medical Chamber Rhineland-Palatinate and the local ethics committees (number 837.376.14). Informed written consent was obtained from all the study participants, and the study has been performed as per the Declaration of Helsinki.

### Measures and variables

The employment status of participants and the frequency of returning to work are the endpoints of interest. Returning to work was evaluated by asking the participants who were working before lung cancer whether they had returned to their job or not after sick leave. Descriptive characteristics of the study population were also assessed. Time to re-employment or retirement (date of re-employment/date of retirement minus date of the first diagnosis) was evaluated in months. Participants were considered “early retired” if they received a full health-related early retirement pension before reaching the age of 65 years.

We were also interested in the intention to returning to work among lung cancer survivors. It was assessed by asking the participants whether or not they had wanted to work again after the cancer diagnosis or not. The literature was reviewed to select the potential independent variables to return to work [5, 10–14]. The selected candidate independent variables for the regression model were gender, age at diagnosis, current or last occupation, level of professional qualification, disease status, use of social service counseling (SSC), UICC stage at diagnosis, comorbidities, emotional functioning, fatigue, monthly household income, types of treatment received, and time since last treatment.

Study questionnaires, patient interviews, and the medical records of the participating hospitals were the sources of data for this study (Fig. 1). The data on the variables “returning to work (yes, no)” and “employment status (employed, unemployed, housewife/husband, disability pension, retired)” were obtained from the patient interviews. The socio-demographical data, disease status, professional qualification, social service counseling support, and time until returning to work were also collected from the interviews. Information on types of treatment received, comorbidities, and other medical data were collected from the clinical records. The European Organization for Research and Treatment of Cancer Quality of Life Core Questionnaire (EORTC QLQ-C30) was used to collect information on emotional functioning and fatigue [21, 22].

## Statistical analysis

### Dealing with missing values

Participants with missing values in the outcome variables were excluded from the analysis. For independent categorical variables, a separate category of missing values, named “unknown,” was created if at least 5 observations had missing values. For the missing items of some variables (emotional functioning, fatigue), mean imputation was done if at least half of the participants had answered. Otherwise, the variable was not used for the regression models. If any category of a variable contained too few participants (< 5), that category was combined with other categories (“radiotherapy only” category of “treatment received” variable was combined with another category “systematic therapy only”). During calculation of the time to returning to work, the missing date was replaced by July 1, if only the associated year of re-employment/retirement and year of first diagnosis were available.

### Dealing with bias and non-participants

A possible selection bias was determined by a comparison of the study participants with the non-participants. Non-participants were considered those who are eligible and contacted but did neither participate in the questionnaire nor the interview part of the study. A detailed non-participant analysis was performed in a previous paper [21].

### Statistical procedures

Descriptive univariate analyses (absolute and relative frequencies, mean values, standard deviation (SD), the proportion of missing values) were performed to examine the frequency of “returning to work,” employment status of participants, time until RTW in months, and study population characteristics. Chi-square test was performed to compare between two groups: RTW and non-RTW.

Associations of independent variables with the outcome RTW (yes/no) were analyzed using binomial multivariable logistic regression, and the strength of associations was expressed as odds-ratios (OR) with 95% confidence intervals (CI). A backward step-wise variable selection was performed. Before performing the regression model, multicollinearity among variables was examined with variance inflation factor (VIF) [23, 24]. Conventionally, VIF with a value of 5 or more indicates a large multicollinearity problem [23, 24].

To identify the influencing factors of the time of RTW, potential candidate variables were entered into a Cox’s proportional hazards model to calculate hazard ratio. The extended Cox model was applied by using time dependent variables to assess proportional hazard (PH) assumptions [25]. If a proportionality test is found to be significant (*p* < 0.05), the proportional hazard (PH) assumption is not satisfied for at least one independent variable in the model [25]. To determine which candidate independent variable did not satisfy the PH assumption, backward elimination was done [25].

The same Cox model was stratified by social service counseling in a second step to assess the association between the use of SSC and time to RTW. Two adjusted survival curves were plotted to compare time to RTW between participants who received social service counseling versus those who did not. The curves were plotted from 7 months after diagnosis and onwards assuming the first few months to be sick leave on average. All statistical analyses were performed using Statistical Analytical Software (SAS) 9.4 version.

## Results

### Descriptive univariate analysis

The study sample is comprised of 232 lung cancer survivors who were < 65 years of age during diagnosis, whose information about RTW was available, and who were employed or working before diagnosis. The mean age was 54.3 years (range 32–64, SD 7), and 52% were male (Table 1).

**Table 1:** Descriptive characteristics of the study sample and subgroups

	Total sample, *N* = 232 *N* (%)	RTW *N* = 113(48.7%) *n* (%)	Not RTW *N* = 119(51.3%) *n* (%)	*p* value (chisq test)
Sex				0.6
Male	120 (51.7)	63(55.8)	57(47.9)	
Female	112 (48.3)	50(44.3)	62(52.1)	
Age at diagnosis				< 0.0001(*t* test)
Mean, range, SD	54.3, 32–64, 7.0	52.8, 32–64, 7.3	55.6, 35–64, 6.4	
Last or current occupational position				0.07
Blue collar worker	46 (19.8)	24 (21.2)	22 (18.5)	
Civil servant	13 (5.6)	7 (6.2)	6 (5.0)	
White-collar worker	145 (62.5)	67 (59.2)	78 (65.6)	
Self-employed	17 (7.3)	11 (9.7)	6 (5.0)	
Unknown	11 (4.8)	4 (3.5)	1 (0.8)	
Types of professional training/qualification				0.5
None	27 (11.6)	11 (9.7)	16 (13.5)	
Vocational training/company training	138 (59.5)	67 (59.3)	71 (59.7)	
Vocational school (master craftsman/technical school, technical academy)	31 (13.3)	14 (12.4)	17 (14.3)	
University (University/University of Applied Sciences/Engineering School/University of Cooperative Education)	31 (13.3)	17 (15.0)	14 (11.8)	
Unknown	5 (2.2)	4 (3.5)	1 (0.8)	
Stable relationship				0.7
Yes	182 (78.5)	90 (79.7)	92 (77.3)	
No	50 (21.6)	23 (20.4)	27 (22.7)	
Monthly household net income				0.06
< 1000€	27 (11.6)	11 (9.7)	16 (13.5)	
1000–2000€	85 (36.6)	33 (29.2)	52 (43.7)	
2000–3000€	51 (22)	25 (22.1)	26 (21.9)	
3000–4000€	27 (11.6)	18 (15.9)	9 (7.6)	
> 4000€	26 (11.2)	17 (15.0)	9 (7.6)	
Unknown	16 (6.9)	9 (8)	7 (5.9)	
Disease status				0.001
Complete Remission	125 (53.9)	74 (65.5)	51 (42.9)	
Remission	14 (6.0)	7 (6.2)	7 (5.9)	
Stable	60 (25.9)	25 (22.1)	35 (29.4)	
Progression	22 (9.5)	3 (2.7)	19 (16.0)	
Unknown	11(4. 7)	7 (6.2)	7 (5.9)	
UICC stage during diagnosis				0.04
Stage I	47 (20.3)	25 (22.1)	22 (18.5)	
Stage II	60 (25.9)	38 (33.6)	22 (18.5)	
Stage III	79 (34.0)	30 (26.6)	49 (41.2)	
Stage IV	39 (16.8)	16 (14.2)	23 (19.3)	
Unknown	7(3.0)	4 (3.5)	3 (2.5)	
Comorbidities				0.7
Yes	128 (55.2)	64 (56.6)	64 (53.8)	
No	104 (44.8)	49 (43.4)	55 (46.2)	
Fatigue (mean, SD)	46.6,29.3	40.9, 29.5	52,29.4	< .0001 (*t* test)
Emotional functioning (mean, SD)	61.8,26.4	66.1,25	57.7, 27.2	< .0001 (*t* test)
Types of treatment received				0.04
Surgery only	49 (21.1)	32 (28.3)	17 (14.3)	
Surgery+ ST+/-RT	103 (44.4)	50 (44.3)	53 (44.5)	
ST only/RT only	19 (8.2)	8 (7.1)	11 (9.2)	
ST + RT	54 (23.3)	19 (16.8)	35 (29.4)	
Unknown	7 (3.0)	4 (3.5)	3 (2.5)	
Time since last treatment				0.04
Currently in treatment or < 1 month	38 (16.4)	16 (14.2)	22 (18.5)	
1 < 12 months	22 (9.5)	5 (4.4)	17 (14.3)	
≥ 12 months	170 (73.3)	91 (80.5)	79 (66.4)	
Unknown	2 (0.9)	1 (0.9)	1 (0.8)	
Use of social service counseling				0.9
Yes	125 (53.9)	61 (54)	64 (53.8)	
No	104 (44.8)	51 (45.1)	53 (44.5)	
Unknown	3 (1.3)	1 (0.9)	2 (1.79)	

*RTW* Returning to work, *chi-sq.* chi-square test, *SD* standard deviation, *UICC* International Union Against Cancer, *ST* systemic therapy, *RT* radiotherapy

#### Occupation

About 67% of 232 participants were not employed at the time of the survey. More than 51% (*N* = 119) of the survivors who were employed before lung cancer diagnosis did not return to work after their diagnosis. Among the working participants, 49% were pursuing a full-time job. Forty-seven percent of the non-employed survivors retired early, and 39% of them had taken disability pension. Only 7% of non-employed participants reported being unemployed. Sixteen percent of employed survivors had changed their job type. The primary reason for non-employment was the physical inability to pursue a profession. Seventy-six percent of the participants had expressed a desire to go back to their job again.

Among the participants who did not return to the job, 66% of them did not return to work even after 4 years post diagnosis (data not shown).

#### Time to RTW

During calculation of time to RTW, for 24 participants, the missing date was replaced by July 1. The mean duration of RTW was around 13 months (mean, 12.7 months; SD, 14.1; 95% CI, 9.9–15.5; SE, 1.4) after diagnosis.

#### Factors possibly associated with RTW

During analyzing different types of profession, white-collar workers were in the highest percentage (62.5%) among other occupation (Table 1). Among various professional training, almost 60% of the survivors had vocational training. The majority of the survivors (78.5%) were in a stable relationship. More than half of the participants (54%) were in complete remission stage. Among different UICC stage, 34% of the total survivors had stage III lung cancer and 26% of the survivors had stage II lung cancer. Presence of comorbidities was common in the participants (55.2%). Mean fatigue score is 46.6 (SD 29.3), and mean emotional functioning score is 61.8 (SD 26.4) among the participants. Most of the patients (44.4%) had received combined surgery, systemic therapy with/without radiotherapy, and 2/3 of total survivors received their last treatment more than 12 months earlier. Almost 54% of patients were benefitted by social service counseling (Table 1).

#### RTW versus non-RTW

The mean age of the survivors who returned to work was 52.8 years (SD, 7.3; 95% CI, 51.5–54.2; SE, 0.7) at the time of diagnosis, and mean age of those who did not return was 55.6 years (SD, 6.4; 95% CI, 54.4–56.8; SE, 0.6). Significant gender differences between RTW and non-RTW were not observed (Table 1). Fifty-nine percent of those who returned to work were white-collar workers. During assessment of monthly household income of the survivors, majority of them (44%) who did not resume work had an income range of 1000–2000 Euro monthly.

About 66% of participants who had returned to work were in complete remission. Three percent of them had progressive disease, and 14% of them were diagnosed with UICC cancer stage IV. The mean symptom score for fatigue was 40.9 (SD, 28.4; 95% CI mean, 35.7–46.3) among survivors who returned to work and 52 (SD, 29.4; 95% CI mean, 46.7–57.3) among those who did not. In contrast, higher emotional functioning score (mean, 66.1; SD, 24.9; 95% CI mean, 61.4–70.7) was observed among patients who could return to work than not returning participants (mean score 57.7, SD 27.1). However, there was no evidence that type of treatment, time since last treatment, or comorbidities showed any notable differences between those who returned to work and those who did not return (Table 1).

### Multivariable logistic regression

Checking multicollinearity, the variance inflation factor (VIF) of all the candidate factors of RTW was below 2 (Supplementary Table 1). After backward variable selection, the following variables remained in the model as independent variables of RTW: age at diagnosis, disease status, UICC stage at diagnosis, fatigue, use of social service counseling, and monthly household net income (Table 2). Every 1-year increase in age at the time of diagnosis decreased the likelihood of RTW (OR = 0.9, 95% CI: 0.88–0.97). During the assessment of the disease status, participants in remission stage were associated with a higher chance of returning to their profession (OR = 8.6, 95% CI: 1.6–57.9) than participants with progressive cancer. Likewise, during comparing the role of UICC stage at diagnosis in RTW, participants who were diagnosed with UICC stage III (OR = 0.5, 95% CI: 0.2–1.2) or stage IV (OR = 0.4, 95% CI: 0.1–1.2) were less likely to return to work compared to the patients at stage I. An increasing trend of RTW was detected with increasing monthly household income (Table 2).

**Table 2:** Predictors of returning to work (multivariable logistic regression)

Independent variables	OR	95% CI	*p* value
Age at diagnosis	0.9	0.88–0.97	0.002
Disease status Remission vs progression Stable vs progression CR vs progression	0.11		
	8.6	1.6–57.9	
	6.1	1.6–31.1	
	6.6	1.8–32.3	
Monthly household net income 1000–2000€ vs < 1000€ 2000–3000€ vs < 1000€ 3000–4000€ vs < 1000€ > 4000€ vs < 1000€	0.07		
	0.8	0.3–2.2	
	1.03	0.3–3.1	
	3.3	0.9–12.1	
	2.4	0.7–9.03	
UICC stage at diagnosis Stage II vs stage I Stage III vs stage I Stage IV vs stage I	0.02		
	1.9	0.8–4.6	
	0.5	0.2–1.2	
	0.4	0.1–1.2	
Fatigue	1.0	0.97–0.99	0.02
Use of social service counseling Yes vs no			0.32
	1.5	0.8–2.8	

*OR* odds ratio, *CI* confidence interval, *CR* complete remission, *UICC* International Union Against Cancer

### Multivariable Cox regression

**Fig. 2:**
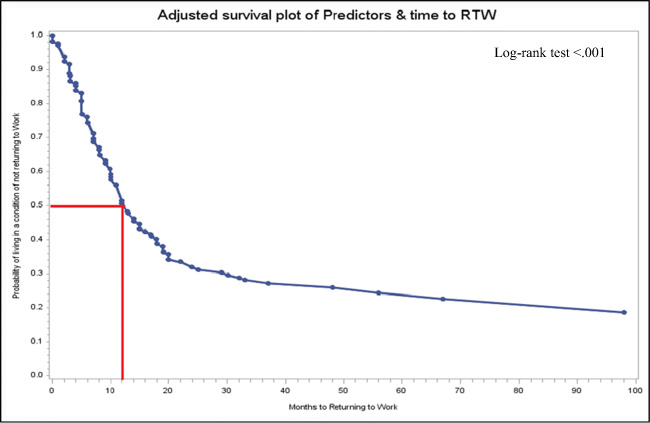
Adjusted probabilities of living in a condition of not returning to work by months since diagnosis

To assess the influencing factors of time to RTW, a multivariable Cox regression model was used. The exact date of RTW was not available in 11 participants who were reinstated in the work force. Among the remaining survivors, influencing variables did not meet the proportional hazard assumption; therefore, backward elimination was done. This resulted in a model including gender, age at diagnosis, types of professional qualification, disease status, fatigue, social service counseling, monthly household net income, types of treatment received, UICC stage at diagnosis, and time since last treatment as influencing variables for RTW (Table 3). The Cox model also showed a decreasing hazard of RTW with female gender (HR = 0.7, 95% CI: 0.5–1.2) and with every 1 year of increase in age during diagnosis (HR = 0.9, 95% CI: 0.9–1). Having a professional qualification acquired at a university level was associated with a 2-fold higher chance of returning to occupational life (HR = 2.2, 95% CI: 0.8–5.8) compared to those who had no qualifications. Regarding the disease status, participants with stable lung cancer showed the highest hazard ratio (HR = 5.6, 95% CI: 1.5–20.3) compared to participants at a progressive stage. Similarly, participants with higher fatigue scores (HR = 0.5, 95% CI: 0.3–0.9) showed low hazard of returning to the job. Complex treatment modalities were associated with very low hazard compared to surgery. The adjusted survival curve of time to RTW (Fig. 2) showed an increasing probability of RTW with increasing months after diagnosis. Within 12 months after being diagnosed with lung cancer, the probability of not RTW was 50%.

**Table 3:** Predictors of time to returning to work (multivariable Cox regression)

Predictors	β	SE	HR	95% CI	*p value*
Gender	0.19				
Male	Ref				
Female	− 0.3	0.2	0.7	0.5–1.2	0.19
Age at diagnosis	− 0.1	0.02	0.9	0.92–0.98	0.004
Types of professional qualification	0.01				
None	Ref				
Vocational training/company training)	0.2	0.4	1.2	0.5–2.7	0.72
Vocational school (master craftsman/technical school, technical academy)	− 0.1	0.5	0.9	0.3–2.4	0.79
University (University/University of Applied Sciences/Engineering School/University of Cooperative Education)	0.8	0.5	2.2	0.8–5.8	0.12
Disease status	0.02				
Progression	Ref				
Not detectable	1.7	0.7	5.4	1.5–19.8	0.01
Remission	1.7	0.8	5.5	1.2–26.2	0.03
Stable	1.7	0.7	5.6	1.5–20.3	0.01
Fatigue	0.06				
Less	Ref				
Medium	− 0.2	0.3	0.8	0.5–1.4	0.41
High	− 0.7	0.3	0.5	0.3–0.9	0.02
Use of social service counseling	0.06				
Not used	Ref				
Used	0.5	0.2	1.6	1.002–2.546	0.048
Monthly household net income	0.21				
< 1000€	Ref				
1000–2000€	− 0.5	0.4	0.6	0.3–1.3	0.20
2000–3000€	− 0.2	0.4	0.9	0.4–1.9	0.7
3000–4000€	0.4	0.4	1.4	0.6–3.4	0.42
> 4000€	− 0.04	0.5	0.9	0.4–2.4	0.92
Treatment type	0.01				
Surgery only	Ref				
Surgery + ST ± RT	− 1.2	0.4	0.3	0.1–0.6	0.0004
RT only/ST only	− 1.2	0.6	0.3	0.1–0.9	0.04
RT + ST	− 1.4	0.5	0.2	0.1–0.7	0.01
UICC stage at diagnosis	0.05				
Stage I	Ref				
Stage II	0.7	0.3	1.9	0.9–3.8	0.06
Stage III	− 0.2	0.4	0.8	0.4–1.9	0.65
Stage IV	− 0.1	0.5	0.9	0.3–2.3	0.79
Time since last therapy	0.13				
Currently in treatment or < 1 month ago	Ref				
1 < 12 month ago	− 1.2	0.6	0.3	0.1–0.9	0.04
≥ 12 months ago	− 0.8	0.4	0.4	0.2–0.9	0.05

**Unknown categories are not reported

*SE* standard error, *HR* hazard ratio, *CI* confidence interval, *UICC* International Union Against Cancer, *ST* systemic therapy, *RT* radiotherapy

### Social service counseling and time to RTW

**Fig. 3:**
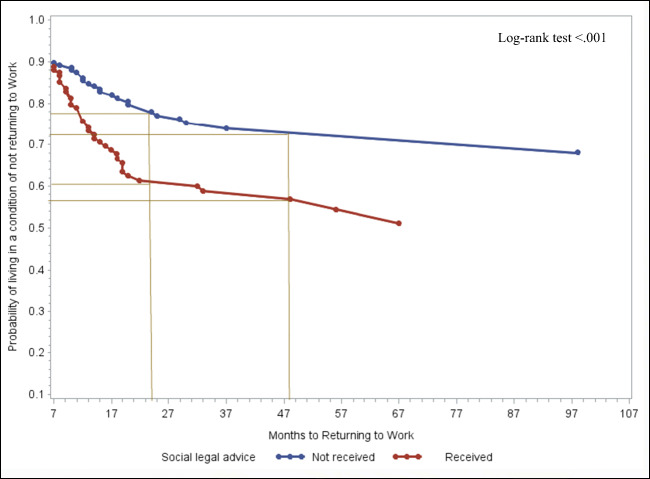
Adjusted probabilities of living in a condition of not returning to work by months since diagnosis and use of social service counseling

Use of social service counseling appeared to facilitate regaining one’s professional life (HR = 1.6, 95% CI: 1.002–2.546, *p* = 0.048) (Table 3). In Fig. 3, patients who had received legal assistance or social service counseling showed a tendency to return to their professional life earlier in comparison to those who did not receive any.

The survivors who did not have any legal assistance showed high probabilities of non-RTW (Fig. 3). In particular, the differences between the two curves widen from 7 months onwards up to 22 months’ post diagnosis. Patients who attended social counseling services showed a 39% probability of having returned to work 2 years after diagnosis, while patients who had not had only a 22% probability of having returned to work at same time point. At 4 years post diagnosis, the probabilities of returning to work for both groups were 44% and 28%, respectively.

## Discussion

Lung cancer is often considered as a disease of older people and is associated with a very low survival rate [26, 27]. Hence, to date, the employment situation of this elderly population has not been thoroughly analyzed. This study has exclusively investigated the employment status of the lung cancer patients of working age in recent times. We found that about two-third of the study sample was not employed at the time of the survey. This result maintains consistency with the results of a previous study on lung cancer survivors, which found that after receiving treatment, 61% were not employed [16]. Our study detected that among the working survivors, only half of them were pursuing a full-time job. This is also similar to the results of Nekhlyudov et al., who reported the need for changing work schedules and reducing workloads among cancer survivors [28]. However, Endo et al. reported a much higher percentage of survivors pursuing a part time job [29].

Abrandt et al. found that the risk of retirement was 1.5-fold larger for the lung cancer survivors compared to their reference group, which is comparable to our study, where almost half of the non-employed survivors retired early [11]. One-third of non-employed survivors were granted a disability pension in our study, which is higher than the work-related disability reported in a Dutch study [30].

Even after years post-diagnosis, the employment situation of lung cancer survivors still remains impacted by lung cancer. We found that among the survivors who did not return to work after their lung cancer diagnosis, 66% of them had not returned to work even 4 years after diagnosis. Likewise Kim et al. (2014) reported that after a median of 4 years after diagnosis, lung cancer patients were more likely to be unemployed than the patients at the time of diagnosis [16].

There appear to be inconsistencies in the mean duration of RTW among studies. In our study, survivors returned to work after an average of 13 months from diagnosis. In a Dutch study, the mean duration of full RTW is reported 343 days in 2008 [31]. On the other hand, the mean duration of sick leave was 150.6 days in another German study [32]. In a Swiss study, it is stated that 63% of lung cancer survivors were still on sick leave even 1 year after diagnosis [33].

Our analysis showed a higher risk of non-RTW with older age, which is also consistent with previous studies [16, 34, 35]. Our study also found an association of disease remission, higher household income, and monotherapy with surgery with a higher chance of RTW. This compares favorably to the findings of an earlier study on lung cancer patients [16]. Fatigue was a frequent symptom reported among those who could not return to job or had job loss in earlier studies [16, 34, 36].

Our study discovered that use of social service counseling (SSC) facilitated the regaining of one’s professional life. We also found that a trend of returning to professional life much earlier among those survivors who attended this service. The routine provision of SSC along with psycho-oncologic care for every patient is one of the fundamental requirements established in the German Cancer Society’s cancer center certification system [37]. This counseling service is contributing to occupational rehabilitation and supports employees who face discrimination at their workplace due to their illness [20]. This has shown to influence the restoration of professional life among lung cancer patients.

The majority of the participants (76%) expressed their desire to go back to their job in this study. Due to the cross-sectional study design, it was measured after returning to work measurement. Due to this temporal problem, it could not be considered as a potential predictor of RTW. Therefore, it was not entered into the regression models. Other study limitations regarding the cross-sectional design of the study are that it prevents determining causality. This study did not include any control group. It is recommended that RTW interventions should be carried out close to the previous workplace in the collaboration of the key stakeholders across different arenas of healthcare systems and social insurance [38–40]. Studies showed adjustments and accommodation of the workplace and consensus between sick-listed employee, and the supervisors have been found to be an important factor for work reintegration among the persons with the musculoskeletal disorder or mental health problems [38, 39]. However, this cannot be implemented for those who do not return to the same job after an illness. Therefore, the information of returning to the same job or not is important in RTW process, since the process could be different and longer if it is not in the same job. It is therefore a clear limitation that we did not collect data ascertaining whether people returned to the same job or not.

Another limitation of this study is that clinical records sometimes lacked updated information and adequate integrity [21, 22]. Multiple imputations of the missing data cause a reduction of the variances of the variables, and thus, relationships between variables were not preserved. Analytical results containing a wide range of confidence intervals of certain variables also create greater uncertainty of the effect size. The generalizability of the study results might be limited, as the employment rate generally depends on social security, welfare policies, and the economic situation of a country. The findings of this German-based study may allow generalizations with other European countries with similar economic structure but not with the other countries of different economic situations.

## Conclusion

This study provides an insight into RTW in those who have undergone treatment for lung cancer. Research on this is sparse, and therefore, the quite large cohort of lung cancer survivors of this study with good response rate provides relevant information. In summary, lung cancer survivors were found to have high risk of unemployment and very low professional reintegration after an interruption due to illness. Social service counseling was found to be influential in returning to professional life. Further studies with follow-up study, more rehabilitation programs, and interventions for patients and healthcare professionals will help to better understand the professional needs of lung cancer survivors.

## Supplementary information

ESM 1 (XLSX 10.6 kb)

## Data Availability

All the data used in this study will be available upon request.
